# The Egosyntonic Nature of Anorexia: An Impediment to Recovery in Anorexia Nervosa Treatment

**DOI:** 10.3389/fpsyg.2017.02273

**Published:** 2017-12-22

**Authors:** Eva C. Gregertsen, William Mandy, Lucy Serpell

**Affiliations:** Department of Clinical, Educational and Health Psychology, University College London, London, United Kingdom

**Keywords:** anorexia nervosa, treatment, recovery, engagement, motivation, egosyntonicity

## Abstract

A serious problem faced by clinicians treating anorexia nervosa is the egosyntonic nature of the illness, wherein individuals with anorexia nervosa value their disorder, thereby hindering motivation for recovery and engagement with treatment. The objective of this review article is to elucidate the nature of egosyntonicity in anorexia nervosa, reviewing both qualitative and quantitative research pertaining to this topic, and, importantly, to present methods to overcome this impediment to recovery in anorexia nervosa treatment. The authors elucidate functions of anorexia nervosa for patients, both within psychological and social spheres, as well as highlight the detrimental effect of egosyntonicity in terms of illness severity and motivation for recovery. The final part of the paper contains suggestions as to methods of bypassing pitfalls linked with the influence of the egosyntonic nature of anorexia nervosa within a treatment setting, as well as an examination of three current treatments (enhanced cognitive-behavioral therapy for eating disorders, Maudsley Model of Anorexia Nervosa Treatment for Adults, and Specialist Supportive Clinical Management) in terms of the degree to which they target egosyntonicity.

## Introduction

Anorexia nervosa (AN) is an illness which is often chronic in nature, wherein sufferers experience a multitude of detrimental effects in various domains of their life as a result of their low weight and restrictive nutritional intake. Considering these negative effects, apparent in physical, mental, and social aspects, it may be tempting to regard the disorder as entirely negative, and as such, AN sufferers’ reluctance to seek, to continue, or to engage with treatment can often be baffling from an outsider’s perspective. Comprehending this apparent paradox of why AN sufferers continue to place value on their disorder despite the array of detrimental effects that comes alongside it may be of significant empirical and clinical importance. In spite of the apparent detrimental effects of the illness, AN treatment is characterized by high drop-out rates, with between 20 and 51% of patients terminating treatment before having seen it to completion in inpatient populations, and 23 to 73% of patients dropping out prematurely in outpatient settings (Fassino et al., 2009). Furthermore, AN patients rarely seek treatment on their own initiative but are often brought to treatment by concerned friends or family members (Freda et al., 2015). At times treatment has to be enforced through the use of mental health legislation. Both treatment drop-out and treatment avoidance highlight the difficulties found in engaging and maintaining treatment adherence in people with AN (Fassino and Abbate-Daga, 2013). In understanding why patients may resist treatment, a number of researchers have identified the egosyntonic nature of AN, that is, the fact that the disorder is often highly valued by those who suffer from it, as a possible culprit in sustaining the illness despite intervention efforts (Serpell et al., 1999).

## Understanding the Egosyntonic Nature of an

Researchers and clinicians have debated how to understand the meaning of anorexic behavior, with some suggesting that AN functions as a way of reclaiming control of psychobiological maturing (Crisp, 1997) or as a self-punishing defense when fearing loss of control (Bruch, 1982). In an attempt to systematically explore the meaning that patients with AN ascribe to their anorexic behavior, a research team based in Norway interviewed 18 women (14 outpatients, 4 inpatients), aged 20 to 34, who had within the past 2 years been diagnosed with AN, utilizing a qualitative thematic analysis to assess the interview data (Nordbo et al., 2006). In a similar study by Serpell et al. (1999), AN patients’ attitudes toward their own illness were assessed by exploring recurrent themes within two letters which patients had been asked to write their anorexia as part of therapy, one addressing their anorexia as a friend and another addressing it as an enemy. The sample comprised 18 patients, all diagnosed with AN (DSM-IV criteria). These studies revealed diverse ways in which AN was experienced as helping the patients achieve goals, or manage difficult experiences.

Thirteen themes relating to the ways in which AN was valuable to the patients emerged, with five of these themes (Security/Control; Avoidance; Mental Strength/Skill; Communication; and Confidence) being common to both studies. Firstly, patients endorsed the notion of AN as a means of achieving a sense of stability and security; by organizing their day through stringent time schedules and rules, the sufferer obtains a sense of structure in their life. Moreover, AN was described as a way of evading negative emotions and experience; when the sufferer’s everyday life is characterized by a myopic fixation on body, food, and weight, there remains little energy to focus on other difficulties or problems. Furthermore, AN was reported as a mechanism by which an inner sense of strength, mastery and skilfulness could be achieved; as the individual with AN loses weight, he or she may experience a sense of mastery and self-control for having managed to adhere to a strict diet and reach weight loss goals. Additionally, feeling worthy of compliments and attaining confidence was reported as a benefit of AN; losing weight makes the sufferer feel attractive and successful, which may be reinforced by positive feedback received externally regarding appearance and dieting performance, thereby increasing self-confidence. Moreover, AN was viewed as a means of communication; a sickly appearance and pathological behavioral patterns may communicate a feeling of distress to others which the sufferer is unable to express with words. Further, themes specific only to Nordbo et al.’s (2006) study described AN as a means of prompting care from others; forging a new, favored identity; and starving oneself to death, in the case where the person does not wish to live anymore, viewing starvation as a less brutal way of dying as compared to suicide. In terms of benefit themes specific only to the work by Serpell et al. (1999), these included feeling special and superior due to having the illness, the loss of periods in instances where this was perceived as positive, and the increased fitness that patients associated with their AN. Of note, a study which employed the same design as Serpell et al. (1999) but within an adolescent population (*n* = 27) demonstrated many similarities and a few notable differences between adolescent and adult AN patients (Freedman et al., 2006). Specifically, compared to their adult counterparts, adolescents tended to place greater value on the sense of feeling looked after by the disorder as well as relished more the increased attention they felt their AN provided them with. In contrast, they did not perceive loss of periods as a benefit.

Furthermore, AN was reported by nine patients as a mechanism by which an inner sense of strength and mastery could be achieved; as the individual with AN loses weight, he or she may experience a sense of mastery and self-control for having managed to adhere to a strict diet and reach weight loss goals. Additionally, eight patients reported that feeling worthy of compliments and acknowledgments was achieved through AN; losing weight makes the sufferer feel attractive and successful, which may be reinforced by positive feedback received externally regarding their appearance and dieting performance, thereby increasing self-confidence. AN was also described by five patients as way of prompting care from other people; as the sufferer becomes sickly looking by virtue of their extreme weight loss, friends and family may express worry and concern, which allows the person with AN to experience the attentiveness, thoughtfulness, consideration, and kindness which they may have previously craved, and to feel cared for by people in their surroundings. Lastly, AN was seen as means of communicating distress to others (*n* = 4), forging a new, favored identity (*n* = 3), and starving oneself to death (*n* = 2), in the case where the person does not wish to live anymore, viewing starvation as a less brutal way of dying as compared to suicide. Serpell et al.’s (1999) study allowed for a deeper understanding of the perceptions of people with AN toward their own illness by also exploring the negative effects of the disorder which patients would identify. The analysis revealed different life domains which the patients perceived their disorder as impacting negatively, as well as negative feelings held toward their illness, demonstrating that despite the egosyntonic nature of AN, patients are still able to recognize various ways in which the disorder causes detriment to themselves, their life, and their loved ones. For instance, patients described elements of feeling cheated or tricked by the disorder in that it had made false or empty promises, as well as feeling anger and hatred toward it and frustrated by constantly having to battle it. In terms of life domains which patients identified as being negatively impacted by the disorder, these included social (including loss of friends, family, social life, and/or career prospects) and health domains. Further, AN was deemed by patients as having a negative impact on emotions in that it made patients feel numb (where this was perceived negatively), as well as leaving them stifled or even taken over by the disorder. Patients also expressed that they were tired of thinking about food incessantly, and that their life and time was being wasted as a result of having anorexia. Lastly, patients identified the psychological symptoms of the disorder such as depressive symptoms as a perceived cost of AN, and also described the upset and hurt which the disorder had caused to family members and friends as a negative effect of the illness. Notable differences between adult and adolescent populations as shown by Freedman et al. (2006) in terms of perceived costs included adolescents describing a higher degree of psychological distress in relation to the illness, and a greater sense of having been fooled by the disorder. Further, adolescents did not describe as much frustration with food preoccupation or being controlled by food.

Of note, upon in-depth reading of the letters, it appeared to the researchers that patients could be grouped into two categories – those for whom attractiveness was an overwhelming motivating factor for AN, and those who completely omitted it when describing its motivators or benefits. Such a finding is of importance in that AN is largely conceptualized within the media as an overzealous attempt at reaching a slim cultural ideal, and in therapeutic contexts wherein this notion is adopted and tackling and debunking beauty myths becomes a highly placed goal within therapy, this goal may have little effect on the patient if attractiveness was not a motivator in developing and/or maintaining their anorexia in the first place. As such, it becomes clear that not only understanding the *degree* to which the egosyntonic nature of anorexia may be an impediment to recovery is of importance in the clinical context, but also dissecting and examining the idiosyncratic meaning anorexia holds specific to each patient. A further point to consider is that some patients who described their anorexia as a method of avoiding emotions or helping to cope with emotions or distress also highlighted the negative theme of anorexia stifling their emotional life, clearly demonstrating the marked ambivalence AN patients often experience in regarding their disorder. This is an ambivalence which can be capitalized upon in the therapeutic context if understood correctly, and also helps explain the indecisiveness AN patients often demonstrate in considering change.

## Perceived Burdens of an

Serpell et al.’s (1999) study allowed for a deeper understanding of the perceptions of people with AN toward their own illness by also exploring the negative effects of the disorder which patients would identify. The analysis revealed different life domains which the patients perceived their disorder as impacting negatively, as well as negative feelings held toward their illness, demonstrating that despite the egosyntonic nature of AN, patients are still able to recognize various ways in which the disorder causes detriment to themselves, their life, and their loved ones. For instance, patients described elements of feeling cheated or tricked by the disorder in that it had made false or empty promises, as well as feeling anger and hatred toward it and frustrated by constantly having to battle it. In terms of life domains which patients identified as being negatively impacted by the disorder, these included social (including loss of friends, family, social life, and/or career prospects) and health domains. Further, AN was deemed by patients as having a negative impact on emotions in that it made patients feel numb (where this was perceived negatively), as well as leaving them stifled or even taken over by the disorder. Patients also expressed that they were tired of thinking about food incessantly, and that their life and time was being wasted as a result of having anorexia. Lastly, patients identified the psychological symptoms of the disorder such as depressive symptoms as a perceived cost of AN, and also described the upset and hurt which the disorder had caused to family members and friends as a negative effect of the illness. Notable differences between adult and adolescent populations as shown by Freedman et al. (2006) in terms of perceived costs included adolescents describing a higher degree of psychological distress in relation to the illness, and a greater sense of having been fooled by the disorder. Further, adolescents did not describe as much frustration with food preoccupation or being controlled by food.

## The Egosyntonic Nature of an and Ambivalence Toward Recovery

### Understanding Ambivalence toward Recovery

In an attempt to understand ambivalence toward recovery in AN, a study investigated the experiences and understandings of those suffering with AN who desired to maintain their eating disorder and examined how these understandings may impact upon treatment experience (Williams and Reid, 2010). Participants (*n* = 14) were recruited via pro-anorexia websites, wherein individuals are encouraged to express positive beliefs about their eating disorder in an online, anonymous environment. Qualitative data was collected and analyzed utilizing IPA. Among identified themes was “Ambivalence and conflict about anorexia.” Ambivalence was expressed as being underlined by the paradoxical notion of AN providing participants with a sense of control, yet as the disease began to overtake their thoughts and behavior, it began to *divest* them of control, rather than provide them with it. This conflict created further ambivalence regarding whether AN was a controllable, functional tool, or a disease that was out of their control and needed to be extinguished. Within the framework of AN being a functional tool, several functions were described, including AN as a way of attaining a sense of strength, achievement, and success, as a coping mechanism, as a way of feeling safe, as an expression of emotion, as an escape from negative situations, affect, and puberty, as a pathway toward happiness, as a tool to punish the self or others, or at its most extreme, as a panacea, with one participant stating, “Thinness will fix everything.” These responses echo the research of Serpell et al. (1999) and Nordbo et al. (2006), further solidifying the notion of the egosyntonic nature of AN as a key component in the maintenance of the disorder. In terms of recovery, marked ambivalence was expressed, with one participant describing both wanting to recover and not wanting to recover in the very same sentence. The potential fatality of AN was highlighted as a reason to strive for recovery. In this vein, it seems that AN patients may desire AN so long as its advantages are perceived to outweigh its disadvantages, and as soon as consequences of AN are considered to drastically outweigh benefits (such as AN becoming life-threatening or taking control over the patient’s life), recovery may become a more appealing prospect. However, one limitation to consider herein is that individuals suffering with AN who seek out pro-anorexic websites, from which this sample was recruited, may hold particularly positive views regarding their illness, which is what fuels them to seek out communication with likeminded individuals in a pro-anorexic online community, and their views may therefore not necessarily be representative of individuals with AN who would not seek out such a community. Nevertheless, the responses found in this study overlap with responses of AN sufferers recruited not from pro-anorexic websites but rather from clinical settings (Serpell et al., 1999; Nordbo et al., 2006).

In another study attempting to elucidate mechanisms behind AN patients’ attitudes toward recovery which employed a descriptive, qualitative design, 36 women (23 outpatients, 11 inpatients, and 2 treatment completers) treated for AN within the past 2 years at four clinical institutions in Norway were interviewed regarding their own perception of what might interfere with their wish to recover (Nordbo et al., 2012). Seven themes surrounding interference with recovery were extracted, with the second highly endorsed theme (*n* = 27) being ‘Appreciating the benefits.’ This referred to a decreased wish to recover as the positive aspects of living with AN were given more weight than, or considered to exceed, the negative aspects. Specifically, patients identified the positive feelings which AN evoked such as feeling secure or having a purpose in life as hindrance to their wish for recovery. Moreover, the high accompanied by being extremely undernourished, the newness and successfulness they felt as a result of one’s AN, the support and care they received from others, and AN being a protected area in which they could always succeed even if failing in all other areas of life, were identified as interfering with motivation for recovery. These findings add a directive subjective link between AN patients’ positive experiences of their illness and the patient’s reluctance toward recovery, supporting the notion that the positive value of AN symptoms comprise a significant contribution to maintaining AN (Vitousek et al., 1998).

### The Egosyntonic Nature of AN and Stages of Change

A framework for understanding ambivalence about change is put forth in the transtheoretical model of change (TTM), wherein individuals are grouped into distinct stages of readiness to change, including precontemplation (not thinking about change), contemplation (thinking about change), action (actively making changes), and maintenance (working to prevent relapse) (Prochaska et al., 1992). Processes of change, referring to the cognitive, affective, and behavioral activities that individuals employ to change problems and work toward new lifestyles, are also described in this model (Prochaska et al., 1988). Among activities of a cognitive nature, decisional balance (DB), defined as identifying and evaluating positive and negative effects of health or risk behaviors, has received special attention. In an integrative study investigating perceived advantages and disadvantages of health-related behaviors across stages of readiness to change, including smoking, quitting cocaine, weight control, reducing high-fat diets, stopping delinquent behavior, safer sex, condom use, radon gas exposure, acquisition of exercise, and physicians’ preventive practice with smokers, it was demonstrated that advancement from precontemplation to contemplation was characterized by an increase of perceived disadvantages, with little change to perceived advantages, in these behaviors (Prochaska et al., 1994). Conversely, progressing from contemplation to action was marked by a decrease in perceived advantages (Prochaska et al., 1994). These two patterns of findings are labeled respectively the strong and weak principles of change.

In an effort to discover the extent to which these findings generalize to eating disorders, differences between anorexics who did not want to change (i.e., precontemplators) and those who were seriously considering recovery (i.e., contemplators) were examined (Cockell et al., 2003). In order to capture these differences, a DB scale developed specifically for AN was utilized (Cockell et al., 2002). This scale comprises three sub-domains; Burdens, Benefits, and Functional Avoidance, wherein Functional Avoidance reflects the use of AN to avoid dealing with other issues, which can be viewed as either a burden or benefit. Results showed that AN patients who were seriously contemplating change identified more costs to the disorder and had greater insight into how it served as a means of avoidance, as compared to those who were not contemplating change, consistent with the strong principle of change. However, the extent to which individuals endorsed benefits of having AN did not vary across pre-action stages of change, failing to support the weak principle of change. Associations between DB scores and symptom severity indices suggested that insight about the function of AN was linked with greater distress.

The study’s findings demonstrate that greater endorsement of the consequences of AN, as well as greater insight into how AN functions as a form of escapism from life’s responsibilities, is linked with more seriously contemplating change, highlighting that patients’ perception of the burdens of their disorder, as well as recognizing the avoidant feature AN brings with it, may play a role in recovery. To move from precontemplation to contemplation, it is possible that recognizing the negative effects of AN is essential. A relevant concept here is the notion of hot versus cold cognition, coined originally to discriminate between cognition mediating processes which are affective in nature (hot) as compared to those which are affect free (cold) (Abelson, 1963). Whilst patients may be able to recognize and acknowledge negatives of AN, an emotional component may be missing, wherein either patients do not feel that these negatives will apply to them as an individual (e.g., despite high mortality rates in AN, the patient does not believe that their AN is severe enough to put them at risk of death), or are simply apathetic to consequences. As such, “feeling” the consequences of AN (e.g., becoming genuinely worried about health risks) as opposed to simply “knowing” them, may be necessary to move from precontemplation to contemplation.

### The Egosyntonic Nature of AN and Illness Severity

Another important question which researchers have attempted to address is whether perceived attitudes toward their illness are linked not only to AN patients’ readiness for change or reluctance toward recovery but to symptom severity. With this aim in mind, Serpell et al. (2004) recruited 233 women with AN and measured eating disorder (ED) symptomatology as well as perceived attitudes toward AN, captured by the Pros and Cons of Anorexia Scale (P-CAN). The P-CAN, developed from the qualitative research by Serpell et al. (1999) described previously, comprises six subscales describing perceived pros of A (Safe/Structured; Appearance; Fertility/Sexuality; Fitness; Communicate Emotions/Distress; and Special/Skill) and four describing cons (Trapped; Guilt; Hatred; and Stifles Emotions). Results demonstrated that strongly held beliefs about the disorder’s positive function was associated with more severe AN, exemplified by a high drive for thinness and greater body dissatisfaction. Of note, AN severity was linked with the pros but not the cons of AN, suggesting that an important distinction between severely ill patients and their less severely ill counterparts is endorsement of the *positive* facets of their illness (Serpell et al., 2004). In this vein, suggestions that tackling the egosyntonic nature of AN may be a key component of therapy for these patients is supported (Treasure, 1999). However, one limitation of this study which arrants mentioning is that diagnostic information was lacking for the majority of the sample, due to participants predominantly having been recruited from a volunteer register as opposed to in a clinical setting. Considering this, the link between perceived benefits and burdens of AN and illness severity may be further elucidated by conducting a similar study within a DSM-V diagnosed sample.

### The Egosyntonic Nature of AN and At-risk Populations

Understanding the egosyntonic nature of AN, and its links to attitudes toward recovery and illness severity, a pertinent question arises regarding the origin of pro anorexic attitudes, specifically, whether such attitudes precipitate the disorder as a risk factor, or develop as an artifact of the illness. With this question in mind, Bailey and Frampton (in preparation) administered the P-CAN in a healthy, albeit high-risk, undergraduate population, asking participants to fill in the P-CAN from the imagined perspective of someone with AN. Results were then compared to P-CAN results from previous AN samples (Serpell et al., 2003, 2004). Contrary to the researchers’ hypothesis, perceived cons of having AN in the healthy sample were significantly lower than the AN adult sample, as well as significantly lower on the con subscales of Guilty and Hatred when compared to AN adolescents, suggesting that someone without AN might not be able to fully comprehend how life-consuming and detrimental the illness can be without having experienced it first-hand. This implies that prevention programs may be served by emphasizing the negative aspects of AN, which a healthy at-risk population may not fully grasp. Furthermore, for the pro subscales Fertility/Sexuality and Communicate Emotions/Distress, the healthy sample expressed less endorsement than AN adults and adolescents, suggesting that these positive perceptions may be disorder-specific, and that endorsement of these perceived advantages may perpetuate the disorder rather than precipitate it. Unexpectedly, however, for the Appearance and Fitness sub-scales, the healthy sample scored higher than the AN samples, indicating that the healthy sample believed that that physical aspects of AN, regarding fitness and appearance, are highly valued by those with AN. This further supports the notion that the general public holds an idea of AN as a disorder mainly concerning the wish to reach an ideal weight and shape, which is misguided, with AN serving functions for sufferers far beyond maintaining or striving toward a certain beauty ideal. Noting this gap between the outsider’s perspective and the AN sufferer’s true experience regarding non-appearance-related maintenance factors is important because it challenges the central role many cognitive theories ascribe to the importance of weight and shape in the cause and maintenance of AN (Garner and Bemis, 1982; Vitousek and Hollon, 1990; Fairburn et al., 1999). A further point to make in this regard is that at-risk populations who view AN as a means of obtaining a perfect appearance may be failing to understand that once ingrained in the disorder increasingly extreme weight loss goals are necessary to obtain satisfaction with one’s appearance, thereby making a “perfect” appearance a goal which can never be reached. Lastly, healthy participants and AN adults scored similarly on the Special subscale, and there were no differences between the healthy sample and AN samples on the Safe/Structured sub-scale. These findings do not contradict research highlighting these aspects as key facets of AN (Serpell et al., 1999, 2004; Gale et al., 2006), but rather imply that perceiving these aspects as positive features of AN is not necessarily disorder-specific. As such, prevention programs, largely currently focusing on body image and body dissatisfaction, may be served by targeting other aspects of AN which at-risk populations regard as positive aspects of the disorder.

## Methods for Overcoming the Egosyntonic Nature of an

Our emerging understanding of specific perceived advantages of AN which patients may endorse and how these weigh up against perceived consequences, potentially playing a role in ambivalence and illness severity, highlights the need to develop methods to counteract this theorized barrier to recovery. Firstly, it is apparent that whilst the egosyntonic nature of AN could be argued to be a core facet of the illness and therefore universal to most AN patients, how this egosyntonic nature presents itself is specific and unique depending on the patient considered, and uncovering this idiosyncratic nature within each patient could be crucial to the therapeutic process. In this vein, psychometric measures developed to capture attitudes toward AN such as the P-CAN might be utilized within a clinical context to give the therapist an overview of which pros and cons of AN the patient endorses and does not endorse, and to work toward finding healthy mechanisms to replace positive functions of AN, as well as solidifying or elaborating negative attitudes already endorsed by the patient. For example, if the patient identifies that AN plays a key function in allowing the patient to evade emotions, the therapist may wish to teach the patient to learn acceptance of the fact that emotions are an unavoidable part of life, or emotion regulation or distress tolerance strategies to employ whenever faced with high emotional distress (such as those which form part of dialectical behavior therapy). Furthermore, if the patient identifies the health risks of AN as a strongly endorsed consequence, the therapist may choose to educate the patient on specific health risks associated with AN as demonstrated by scientific evidence, in order to further ingrain this endorsement. Something to be considered, however, in this regard is the patient’s stage of change. According to research investigating the TTM, wherein a sample of smokers were examined, results suggested that precontemplators process less information about their negative behavior as well as experience fewer emotional reactions to the negative aspects of the behavior targeted for change (Prochaska and DiClemente’s’s1983). Extrapolating these concepts to AN, this may suggest that introducing information on health risks to AN patients who are in the precontemplation stage of change may be futile, as these patients may not be ready to fully process information which is incongruent with the idea that their illness is ultimately beneficial to their life, rather than predominantly detrimental to it. In Prochaska and DiClemente’s (1983) research, contemplators, on the other hand, proved to be the most likely to be responsive to feedback and education as sources of information about their behavior targeted for change. As such, considering not only which particular benefits and consequences of their illness AN patients endorse and do not endorse – but also wherein they fall on the spectrum *overall* in terms of readiness to change – seems pertinent in the development of suitable intervention efforts.

Another point to consider regarding the egosyntonicity of AN are how the functions of AN may either overlap or be in dissonance with the patient’s values, wherein values can be defined as things which people place a strong sense of meaning or importance unto (Hayes et al., 2006). For example, adhering to a strict and extreme diet may align with a patient’s value of pushing oneself to extremes in striving for perfection, yet overarching personal values – including those of a spiritual, interpersonal, or career-related nature – may resultantly be neglected or compromised in striving to achieve AN-related goals, highlighting once more internal contradictions which AN sufferers may experience whilst deciding whether their disorder is overall beneficial or detrimental and whether recovery is a desired outcome. In line with this, a qualitative study was conducted exploring personal values among individuals with AN, with a particular focus on the overlap between participants’ values and their AN (Mulkerrin et al., 2016). Themes were identified through Interpretive Phenomenological Analysis (Smith and Osborn, 1999). Among emerging themes, “perceived congruence” and “perceived incongruence” demonstrate how participants experienced a mixed fit between AN and their personal values, with AN both clashing and converging with values which they internalized. Regarding perceived congruence, participants described how AN was experienced both physically (through extreme thinness) and behaviorally (through severe restraint and dietary restriction) as a manifestation of some of their core values, including will power, achievement, hard work, discipline, and self-control. Conversely, regarding perceived incongruence, participants described both AN’s sabotaging effect on their ability to act in accordance with their values, as well as the paradoxical experience of AN seemingly being aligned with their values yet in reality compromising them. Most prominent among values experienced as incongruent with AN were interpersonal values, such as having close friendships and family relationships, caring for others, and having fun and socializing, which were described as being overtly compromised by AN. Further, AN was described as both a means of attaining control but also a means through which the sufferer spirals *out* of control, highlighting its paradoxical nature. Additionally, and perhaps unsurprising given the aforementioned experiences of AN both converging and clashing with personal values, themes were also identified wherein participants expressed both ambivalence toward AN and ambivalence toward recovery. Regarding ambivalence toward AN, participants described experiencing an internal battle in that AN was a safe and familiar option, similar to the ‘Guardian’ theme of Serpell et al.’s (2004) research, yet simultaneously a tormenting and dangerous presence. Distinct from this, participants’ accounts of the ambivalent feelings they held toward recovery seemed underlined by wanting to keep certain valued aspects of AN (for example, increased confidence and more positive body image due to extreme thinness) whilst letting go of all egodystonic symptoms, providing support for the role of endorsement of pros of AN in maintaining the disorder. Lastly, a final theme identified which holds important clinical implications in the attempt to overcome the egosyntonic nature of AN was the theme of “values as a beacon of hope,” wherein participants described focusing on non-eating disorder values as a tool for addressing ambivalence and facilitating motivation to change. Thus, through value-clarification and uncovering personally held values which are incompatible with AN behaviors (for example, placing importance on friendship and spending time with friends yet having to avoid social situations because of AN due to food being involved), patients may be enabled to see how overarching personal values are being compromised by their disorder, and how ultimately if they want to live a life truly aligned with their core values, it is necessary to move on from their eating disorder.

Recognizing and understanding the egosyntonic nature of AN not only has clinical implications related to tailoring treatment but also pertaining to how less ‘compliant’ patients may be viewed by clinicians, which can affect therapeutic outcomes. Often, AN patients who demonstrate high treatment resistance are labeled non-compliant, and may eventually be subjected to staff-initiated discharge from treatment on the basis of not complying with treatment plans or failing to reach target weights. However, if clinicians keep in mind the demonstrated and robust egosyntonic nature of the disorder, with many patients viewing their illness as an accomplishment rather than an affliction (Casper, 1982), non-compliance might almost be *expected*, and should be considered an actual *symptom or artifact* of the illness, rather than some sort of malfeasance or misconduct on part of the patient. As such, a useful paper by Vitousek et al. (1998) on enhancing motivation for change in treatment-resistant eating disorders emphasized the importance of appreciating the degree to which the desire for thinness and self-control is egosyntonic. Furthermore, the authors highlighted the problematic nature of ‘attaching surplus meaning to resistance’ toward recovery. Resistance is to be expected given that the therapist is ‘proposing to fix one of the few domains of the person’s life which they do not regard as broken.’ As Vitousek et al. (1998) suggest, “It is professionally irresponsible to be offended when anorexic patients act anorexic, just as it would be to interpret an obsessive-compulsive client’s hand washing as a personal affront.” The clinical danger here is in labeling patients as non-compliant and therefore not suited for treatment when their resistance toward treatment could be considered natural and even somewhat logical upon regarding the great value which patients perceive their disorder as providing them with, as well as personal values which patients perceive their AN as aligning with. Reframing resistance as a comprehensible response to threat gives both therapists (Garner and Bemis, 1982) and patients (Russell and Meares, 1997) an alternative narrative that may promote empathy and decrease conflict. This reframe might be done by therapists being aware of and acknowledging the functional role which AN may play in the individual’s life (Vitousek et al., 1998; Gremillion, 2003).

## Current Treatments

With respect to current treatments, the degree to which they target the ambivalent nature of the illness varies across treatments. Currently, the main psychological treatments for AN include enhanced cognitive-behavioral therapy for eating disorders (CBT-E), Maudsley Model of Anorexia Nervosa Treatment for Adults (MANTRA), and Specialist Supportive Clinical Management (SSCM). Regarding CBT-E, often considered the mainline treatment for eating disorders, a transdiagnostic approach is adopted, built on the premise that all eating disorders share two core psychopathologies; (1) over-evaluation of weight and shape, and (2) the control of weight and shape. Whilst over-evaluation of weight and shape certainly can be argued to be a hallmark of AN, both quantitative and qualitative research into the egosyntonic nature of AN demonstrate that AN serves as a multi-faceted functional tool for sufferers, and as such, the functional nature of AN in patients’ lives may be far more complex than the illness simply being a means of attaining a thin body coveted as a result of overvaluing weight and shape. Whilst CBT-E does incorporate narratives wherein pros and cons of being underweight are explored, for example through letter writing addressing AN as both a friend and an enemy, thus targeting ambivalence, such activities are usually not proposed until Stage 3 of therapy (sessions 10–17) wherein the main maintaining mechanisms of the eating disorder are addressed. At this point, psychoeducation about the risks of eating disorders and prescription of meal plans and homework may have gone on deaf ears to unmotivated candidates whose concerns about letting go of a central part of their identity and their best discovered coping mechanism to date have yet to be addressed.

Showing a radical departure from classical CBT, MANTRA, a cognitive-interpersonal treatment for AN, has been built on the basis of a cognitive-interpersonal maintenance model which posits that AN typically arises in sensitive/anxious and perfectionistic/obsessive individuals, and is maintained by four broad factors: (1) a rigid, detail-focused, and perfectionistic information processing style; (2) impairments in socioemotional domains; (3) pro anorexic beliefs; and (4) high levels of expressed emotion or accommodation and enabling in close others, with starvation intensifying all these problematic factors. The model is contrasted to CBT-E in that it departs from the notion that weight and shape concerns are *the* central psychopathology of the disorder, arguing that although patients with AN mainly worry about aspects of their ED, these worries are simply symptomatic of more profound and troubling problems. The proposition that weight and shape concerns are not necessarily the core psychopathology of AN is consistent with the previously cited research of Serpell et al. (1999), as patients described not only controlling their weight and shape as the sole central benefit of their illness, but rather endorsed a range of beneficial aspects, most commonly benefits pertaining to attaining safety and structure from AN rather than a desired appearance. Furthermore, in the work of Nordbo et al. (2006), mention of appearance was conspicuously missing, although patients did report feeling more worthy of compliments which may have been appearance-related. As MANTRA is developed based on a model incorporating pro-anorexic beliefs as one of its four key maintenance factors, treatment serves to target the ambivalent nature of the illness in many ways. Firstly, the clinician takes a warm and empathic, reflective, responsive, and collaborative position, in the style of motivational interviewing (Miller and Rollnick, 2002), rather than warning, lecturing, or even threatening patients regarding health risks of their illness, which may make patients “defend” their AN. Furthermore, in the early phase of treatment (sessions 1–4), pro-anorexic beliefs are addressed and motivation for change is built. Patients’ pro-anorexia beliefs which ascribe the illness its own unique meaning are explored through therapeutic writing exercises wherein the value of AN in the person’s life is investigated and questioned. As a helpful addition, patients’ personal values and how AN has changed these are also addressed.

Regarding SSCM, originally developed as a control treatment in a CBT and interpersonal therapy for AN trial, wherein surprisingly its efficacy was equivalent to that of CBT, the therapy’s central focus is on symptoms, with the main aim of therapy being the resumption of normal eating. Whilst a behavioral approach wherein clear goals are set and antecedents for problem behaviors are examined is helpful in terms of teaching AN patients when they are at risk for engaging in eating disordered behaviors and how to combat such situations, failure to explore the egosyntonicity of AN and thus the patient’s motivation may leave the patient with a series of helpful tools that they simply don’t care to use, as a result of still being in the contemplative stage of change. Furthermore, the psychological mechanism of symptoms is left unexplored in this type of therapy. If psychological mechanisms for symptoms are not understood (for example, the patient derives a feeling of significance from being at an extremely low weight and engaging in restrictive dieting which most others simply could not sustain), then the core psychopathology (feeling insignificant) remains unaddressed, and the patient will be more likely to re-engage with eating disordered behaviors in order to obtain the perceived benefit of AN to tend their underlying wound. Whilst SSCM does incorporate psychoeducation, which may solidify patients’ endorsement of cons of AN, it is hypothesized based on preliminary evidence as well as the model upon which MANTRA was built, that it is patient’s endorsement of the *benefits* of AN that is a crucial factor in maintaining the disorder. Thus, emphasizing the downfalls of AN without exploring and dismantling the patient’s perceived benefits may be of limited use. Furthermore, a myopic focus on eating disorder behaviors as opposed to the *meaning* of these behaviors may leave the patient feeling unheard; as such, exploring AN’s egosyntonicity is not only important in terms of finding healthy mechanisms through which the patient can derive that which he or she previously derived from AN, but also because it allows the patient the opportunity to express the unique significance which AN holds for them, facilitating understanding and rapport between patient and clinician.

## Author Contributions

EG conceived of the presented idea. LS and WM supervised the work. All authors discussed results and contributed to the final manuscript.

## Conflict of Interest Statement

The authors declare that the research was conducted in the absence of any commercial or financial relationships that could be construed as a potential conflict of interest.
